# Mercury contamination alters soil microbial communities and functional traits in farmland soils of a mining region, south-western China

**DOI:** 10.3389/fmicb.2025.1721310

**Published:** 2025-12-18

**Authors:** Gao Yu, Fen Chen, Xiaodong Zhang, Zuhua Wang

**Affiliations:** 1 College of A&F Engineering and Planning, Tongren University, Tongren, Guizhou, China; 2 College of Resources and Environmental Engineering, Guizhou University, Guiyang, Guizhou, China

**Keywords:** adaptation mechanism, community structure, functional metabolic traits, mercury mine, soil bacteria

## Abstract

**Introduction:**

This study aimed to determine the status of soil mercury (Hg) contamination and to understand the associated soil microbial community structure and function, along with their relationships with environmental factors, in farmlands surrounding mercury mining regions.

**Methods:**

Soil samples were collected from farmland surrounding a mercury mining region (Chuandong town, CD, Huaqiao town, DP, Bahuang town, BG, and Shuangjiang town, LT) in Tong Ren, south-western China. We analyzed soil physicochemical properties, Hg pollution indices, and bacterial community structure and function. The interactions among soil environmental factors and bacterial community structure and function were determined using correlation analysis and redundancy analysis.

**Results:**

The soils exhibited varying degrees of Hg contamination: CD and LT soils were categorized by “light” Hg contamination, whereas DP and BG soils exhibited “moderate” Hg contamination. The potential ecological risk was “moderate” for CD soils, “considerable” for BG and LT soils, and “high” for DP soils. Long-term Hg contamination significantly increased soil bacterial community diversity and decreased bacterial community richness. Bacterial communities underwent adaptive restructuring, with Acidobacteria (16.90% relative abundance) dominating the acidic, high-Hg soils at the DP site and Proteobacteria (29.71% relative abundance) thriving in nutrient-rich conditions at the LT site. Key metal-resistant genera (*Rokubacteriales*, *Gaiella*) emerged as potential biomarkers of contamination. PICRUSt2 analysis revealed maintained metabolism potential under Hg stress, with carbohydrate metabolism and amino acid metabolism pathways collectively accounting for 26.43% of all predicted functions. Redundancy analysis identified soil pH, THg, and *Gaiella* were the key the factors driving the soil bacterial community function, with their independent contributions contributions to the variance being 72.83, 84.64, and 81.97%, respectively.

**Discussion:**

These findings provide a mechanistic understanding of microbial resilience in Hg-contaminated ecosystems and identify critical leverage points for remediation strategies targeting both metal toxicity and the functional restoration of agricultural soils.

## 1 Introduction

Mercury (Hg) contamination remains one of today’s most pressing environmental crises, posing a serious threat to ecosystem integrity and food security (Grace et al., 2023). As the third most toxic heavy metal, Hg pollution is particularly widespread in China’s Guizhou Province, where the historic Tongren mercury mine-once one of the country’s largest production centers-has left a devastating environmental legacy (Luo et al., 2025; Wu et al., 2020). Despite the cessation of mining activities in 2001, the region continues to struggle with extensive contamination, including the release of an estimated 20.24 billion m^3^ of Hg — contaminated waste gas, 51.92 million m^3^ of wastewater and 4.26 million m^3^ of slag (Teng et al., 2005). The ecological consequences are staggering: soils near smelting sites have Hg concentrations as high as 2,920 mg·kg^−1^ — a staggering 26,545-fold increase over Guizhou’s Province soil background levels (0.11 mg·kg^−1^) (Zhang et al., 2024). The surrounding farmland is also severely contaminated, with an average Hg concentration of 6.00 mg·kg^−1^, exceeding both the Guizhou’s Province soil background level by 54.5 times and the Soil Environmental Quality Risk Control Standard for Soil Contamination of Agricultural Land (GB 15618–2018) by 2.5 times (Tang et al., 2021). In addition, Hg concentrations in key crops, such as rice, maize, pepper and beans, consistently exceed the Chinese food safety limits (GB 2762–2022) (Tang et al., 2021). However, current studies primarily focus on farmland soils near mercury mine (Du et al., 2025). The Hg concentrations and the associated risks in soils on distant farmland remain unclear.

Compared with plants and soil fauna, soil microorganisms, which are critical components of terrestrial ecosystems (Aqeel et al., 2023), are more sensitive to heavy metal contamination and respond rapidly through physiological and community — level adaptations (Naseem et al., 2024). This responsiveness positions microbial ecological traits, such as enzyme activity, respiration rates, community structure and functional diversity, as key indicators for assessing soil contamination (Bhaduri et al., 2022; Khalid et al., 2023; Minnikova et al., 2023). In particular, heavy metal exposure drives dynamic shifts in microbial populations: sensitive taxa decline while metal — tolerant strains dominate, restructuring community composition (Kaur et al., 2024; Pal et al., 2021). However, these effects vary with metal type; for example, Hg strongly alters community structure and function but minimally affects growth rates (Frossard et al., 2018), whereas Cd and Cu significantly affects functional diversity of microbial communities, and Pb has negligible toxicity (Soares et al., 2024; Teng et al., 2005). Notably, research specifically addressing mercury pollution remains insufficient. While existing studies have documented structural shifts in soil microbial communities near mining areas (Du et al., 2025; Liu et al., 2014), the response mechanisms of microbial functional metabolism lack systematic investigation. This is particularly evident in the absence of comparative analyses across different regions or habitat types. Moreover, the adaptation mechanisms of microbial communities in agricultural soils distant from mining sites, with respect to functional metabolic regulation under mercury stress, constitute a critically understudied area. Furthermore, soil physicochemical properties (e.g., pH, organic matter, moisture) modulate metal bioavailability and microbial responses, sometimes outweighing metal concentrations themselves (Jiang et al., 2016; Song et al., 2018). Despite progress, the interplay between metal speciation, soil properties and microbial dynamics remains unresolved, particularly in terms of quantifying their relative contributions to community shifts (Kenarova et al., 2014). Filling this gap is essential for accurate assessment of soil quality. Advances in molecular tools — functional genomics, microarrays, and *in situ* activity assays (Deng et al., 2023) — now provide unprecedented opportunities to unravel these relationships, paving the way for mechanistic insights into microbial resilience and remediation strategies in contaminated ecosystems. This study investigated these relationships in mercury - contaminated agricultural soils across a spatial gradient extending from the Tongren mining region, where long-term Hg pollution has created an ideal natural laboratory. We integrated comprehensive analyses of soil physicochemical properties, Hg concentrations and bacterial community characteristics across contamination gradients to address three key objectives: (1) quantifying the current Hg contamination status, (2) characterizing associated changes in bacterial community structure and functional metabolic traits, and (3) elucidating relationships between environmental factors and microbial responses. By applying advanced molecular and statistical approaches, we aimed to provide mechanistic insights that bridge the gap between metal contamination patterns and their ecological consequences, ultimately informing more accurate soil quality assessments and remediation strategies.

## 2 Materials and methods

### 2.1 Overview of the study area

The study area, situated in Tongren City, Guizhou Province, China. Tongren City is located in the northeastern part of Guizhou Province, in the hinterland of the Wuling Mountains, east of Hunan Province, whereas Huaihua city, north of Chongqing Municipality, has high elevation in the northwest and low elevation in the southeast. The whole territory is dominated by mountains, and most of the territory is in a mid-subtropical monsoon humid climatic zone. The sampling points in this study cover four town in Tongren city: Chuandong town (CD), Huaqiao town (DP), Bahuang town (BG) and Shuangjiang town (LT). The crops that were cultivated in the year under study were *Mesona chinensis*, which were planted in 1a. The natural profile of the sampling points is delineated in Table 1.

**Table 1:** Study area survey.

Sample	Location	Longitude (°E)	Latitude (°N)	Elevation/m	Average annual sunshine /h	Average annual temperature /°C	Average annual precipitation /mm	Distance to Hg mine/km
DP	Huaqiao Town	108°19’50”	27°39’39”	879	871	17.1	1,073	87.88
LT	Shuangjiang Town	108°48’05”	27°39’39”	506	1,257	16.2	1,370	43.92
CD	Chuandong Town	109°12’46”	27°47’16”	458	1,403	15.3	1,100	29.67
BG	Bahuang Town	109°01’22”	27°42’58”	293	1,170	16.8	1,337	28.54

“Hg mine” refers to the Wanshan Mercury Mine, once the largest mercury mine in Asia, located in Tongren.

### 2.2 Soil sample collection

During the harvest period of cool grasses, five sampling points were established in each study area at random, and a sample square measuring 2 m × 2 m was set up at each sampling point. The five-point method was employed to collect soil samples from a depth of 0–20 cm within each sample square, following the removal of the layer of fallen litter and any plant roots, stones or other debris. The soil samples from the same study area were then thoroughly mixed and homogenized, after which they were placed in sealed bags and transported to the laboratory in a timely manner. The samples were stored in a refrigerator at −80 °C for high-throughput sequencing and air-dried for the determination of soil physicochemical indices.

### 2.3 Determination of basic soil physicochemical properties

Soil pH was measured at a soil:water ratio of 1:2.5 (w/v) using a pH meter. Soil organic matter (SOM) content was determined using the K_2_Cr_2_O_7_ oxidation titration method (Meng et al., 2021). The total nitrogen (TN) content of the soil was measured via an automatic azotometer (SKD-100, Peiou Analytical Instrument, Shanghai, China). The available nitrogen (AN) and available phosphorus (AP) contents of the soil were determined with a UV–VIS spectrophotometer (Meng et al., 2021). The available potassium (AK) content of the soil was measured by a flame photometer (Bao, 2008).

Soil total Hg (THg) and effective state Hg (HCl-Hg) were extracted by aqua regia (1 + 1) digestion and 0.1 mol·L^−1^ HCl leaching, respectively, and were determined via inductively coupled plasma–mass spectrometry (ICP-MS, 7700x, Agilent Technologies, Japan). All reagents were of analytical grade, and ultrapure water was used throughout the experiments. All laboratory glassware was soaked in a 15% (*v/v*) nitric acid solution for at least 48 h prior to use. Furthermore, quality control during the analytical procedures was assured through the use of blank samples, 10% duplicate samples, and the national standard material GBW 07401 for soil composition analysis.

### 2.4 Assessment of contamination and ecological risk

The geoaccumulation index method (*I*_geo_) (Muller, 1969) and Hakanson’s potential ecological risk index method (*E*) (Hakanson, 1980) were used to evaluate the contamination status of soil with respect to Hg, as calculated by Equations 1 and 2, respectively.


(1) $$I_{\mathrm{geo}}=\log_{2}(\frac{C}{\mathrm{KS}})$$

(2) E=T(C/S)

where *I*_geo_ is a geochemical index used to evaluate pollution levels in soils or sediments and has been used since the 1960s (Muller, 1969); *C* (mg·kg^−1^) is the measured concentration of soil heavy metals; the coefficient *K* = 1.5, which is used to detect very small anthropogenic influences (Doabi et al., 2019); *S* (mg·kg^−1^) is the background concentration of the element in the soils of Guizhou Province (*S*_Hg_ = 0.11 mg·kg^−1^); *E* is the ecological risk factor for heavy metals; and *T* is the toxic-response factor (*T*_Hg_ = 40). *I*_geo_ of different ranges can be divided into seven grades, i.e., Uncontaminated (*I*_geo_ ≤ 0), Uncontaminated to moderately contaminated (0 < *I*_geo_ ≤ 1), Moderately contaminated (1 < *I*_geo_ ≤ 2), Moderately to heavily contaminated (2 < *I*_geo_ ≤ 3), Heavily contaminated (3 < *I*_geo_ ≤ 4), Heavily to extremely contaminated (4 < *I*_geo_ ≤ 5), and Extremely contaminated (*I*_geo_ > 5). *E* of different ranges can be divided into five grades, i.e., Low risk (*E* < 40), Moderate risk (40 ≤ *E* < 80), Considerable risk (80 ≤ *E* < 160), High risk (160 ≤ *E* < 320), and Very high risk (*E* ≥ 320).

### 2.5 DNA extraction and Illumina MiSeq sequencing

DNA was extracted from the soil samples using a soil DNA kit (OMEGA M5635-02, USA), and the concentration and quality of the DNA were subsequently determined using agarose gel electrophoresis and a fluorescence spectrophotometer (Quantifluor-ST fluorometer, Promega, E6090; Quant-iT PicoGreen dsDNA Assay Kit, Invitrogen, P7589) to determine the concentration and quality of the DNA. Universal bacterial primers (338F: 5’-ACTCCTACGGGAG GCAGCA-3’; 806R: 5’-GGACTACHVGGGTWTCTAAT-3’) were used to amplify the V3-V4 region of the 16S rRNA gene via PCR amplification. The PCR products were purified, quantified and homogenized on the basis of 16S rDNA sequences to construct libraries. Quality control was performed via LabChip, and the quality-checked libraries were sequenced via an Illumina MiSeq platform. The raw sequences were subjected to primer removal, quality filtering, denoising, splicing and dechimerization using DADA2 and finally clustered according to 100% similarity on the basis of the Silva database (Release 132^1^) for bacterial species annotation. High-throughput sequencing of soil bacteria was performed by Shanghai Parsonage Biotechnology Co.

### 2.6 Statistical analysis

Excel 2013 and SPSS 25.0 were used to process the experimental data, and one-way ANOVA and multiple comparison procedures were used to analyse the significance of differences (SPSS 25.0). LEfSe was used to analyse significantly different species of bacteria at five taxonomic levels from phylum to genus (LDA score > 3.6, *p* < 0.05). An undirected correlation network was constructed via the igraph package in R language, the symbiotic network of soil bacteria was mapped via Gephi 0.9.2, and the network topology indices of the soil bacteria were calculated to analyse the differences in the topological characteristics of the soil bacterial networks in the four study areas. Bacterial community function prediction was carried out using PICRUSt2, and sequencing data were compared via the Kyoto Encyclopedia of Genes and Genomes (KEGG) database. STAMP software was used to compare the differences in the abundance of functional genes of soil microorganisms in different regions. Correlation analysis and redundancy analysis (RDA) were conducted via the Psych package, ggplot 2 package and vegan package to explore the relationships among soil environmental factors, bacterial community structure and bacterial community function; assess the relative contributions of soil environmental factors and bacterial community structure to bacterial community function; and determine the main factors controlling bacterial communities.

## 3 Results

### 3.1 Physicochemical factors and Hg contamination status of agricultural soil

#### 3.1.1 Physicochemical indicators

**Figure 1:**
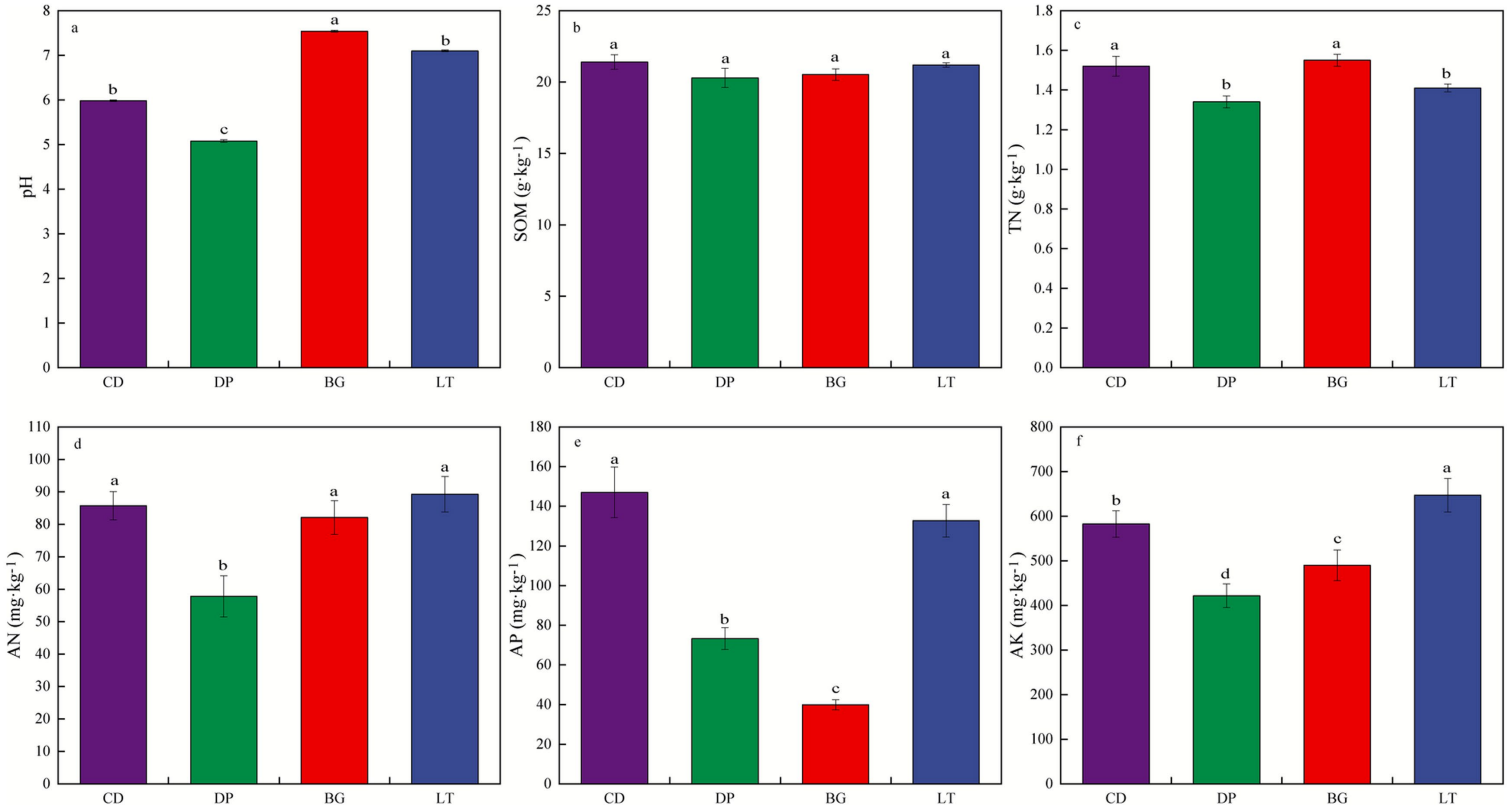
Physical and chemical properties of the experimental soil. **(a)** pH, **(b)** soil organic matter (SOM), **(c)** total nitrogen (TN), **(d)** available nitrogen (AN), **(e)** available phosphorus (AP), **(f)** available potassium (AK). Different lowercase letters represent the significant difference (at 0.05 level, one-way ANOVA test) between different study areas, the same as below.

Figure 1 shows the physical and chemical properties of the experimental soil. Soil pH varied significantly between the study areas (*p* < 0.05), with pH values in the BG and LT ranging from 7.10 to 7.54, which were slightly alkaline, and pH values in the CD and DP ranging from 5.08 to 5.98, which were slightly acidic. The soil organic matter (SOM) content in CD was 5.52, 4.34, and 1.04% higher than in DP, BG, and LT, respectively; however, these differences were not statistically significant. In contrast, the total nitrogen (TN) content in BG was significantly higher than that in LT and DP by 9.93 and 15.67%, respectively (*p* < 0.05). Although the difference compared to CD was not statistically significant, the content in BG was still 1.97% higher. The available nitrogen (AN) content in LT was 54.33% higher than in DP (*p* < 0.05). Compared to CD and BG, the AN in LT remained 4.17 and 8.70% higher, respectively, though these differences were not significant. For available phosphorus (AP), the content in CD was significantly higher than that in DP and BG by 100.50 and 268.86%, respectively (*p* < 0.05). Although the difference between CD and LT was not statistically significant, the content in CD still represented a 10.76% increase. Finally, the soil available potassium (AK) content differed significantly across all four regions (*p* < 0.05). Specifically, the AK content in LT was significantly higher than that in CD, DP, and BG by 9.97, 53.33, and 32.00%, respectively.

#### 3.1.2 Characterization of soil mercury contamination and environmental risks

**Figure 2:**
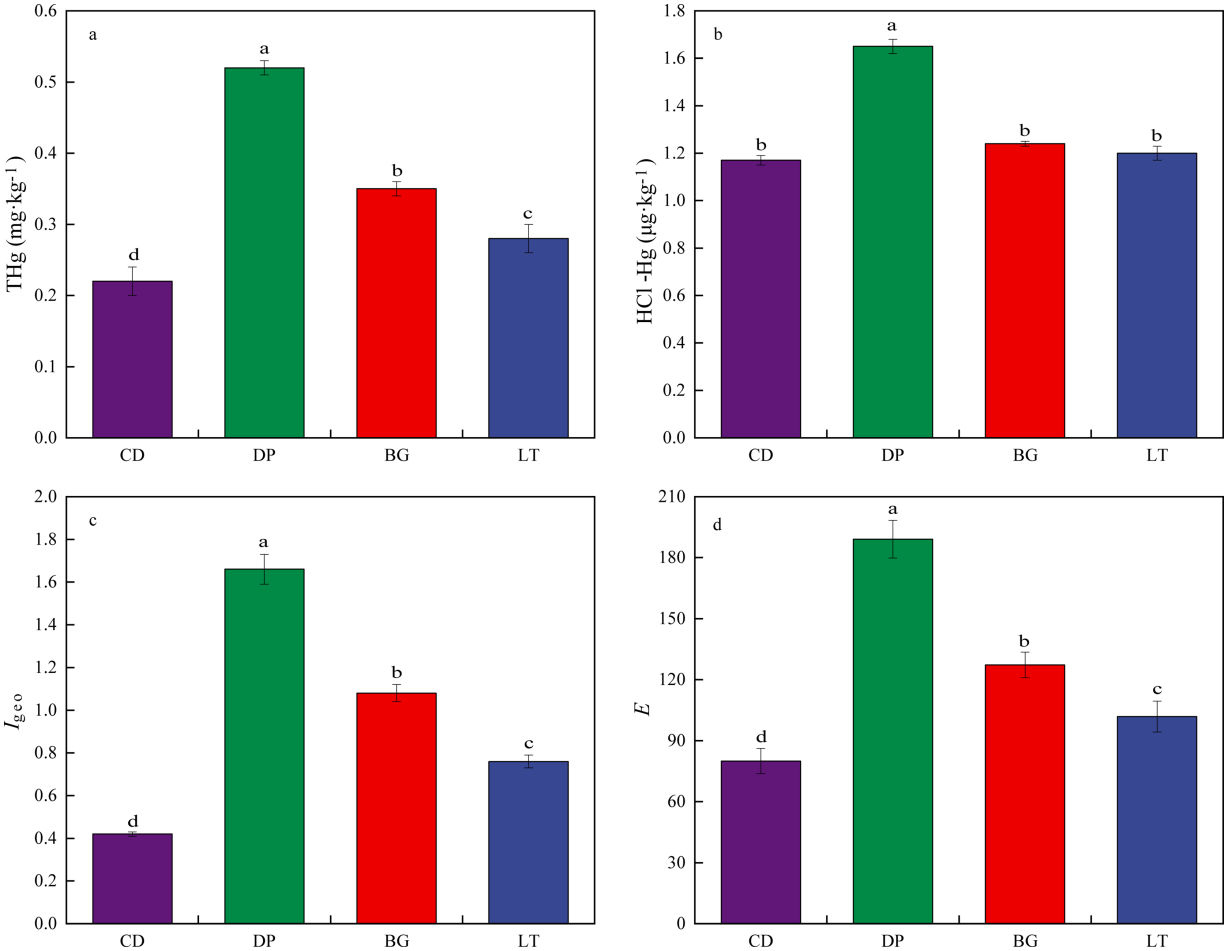
Soil Hg contamination characteristics and environmental risks. **(a)** Total Hg (THg) concentration, **(b)** HCl-extractable Hg (HCl-Hg) concentration, **(c)** Geoaccumulation index (Igeo), **(d)** Potential ecological risk index (E).

Figure 2 shows the data for soil Hg contamination characteristics and environmental risks. The THg concentration of the soil in the study area was 100% ~ 136.36% higher than the background concentration in the soils of Guizhou Province (0.11 mg·kg^−1^). The soil THg and HCl-Hg contents in different areas were different, and the THg and HCl-Hg concentrations were in the order of DP > BG > LT > CD (Figures 2a,b). The soil HCl-Hg concentration is closely related to the THg concentration, and the contamination status of THg can also reflect the contamination status of HCl-Hg. Thus, it was important to clarify the contamination status of soil Hg in the study area to provide theoretical support for the safe production of local food crops. Figures 2c,d show that the differences in the *I*_geo_ and *E* indices of soil Hg in different regions were significant (*p* < 0.05). According to the criteria listed in *I*_geo_, CD and LT were considered uncontaminated to moderately polluted; and DP and BG were considered moderately polluted. According to the criteria listed in *E*, CD posed moderate potential ecological risk. BG and LT posed considerable potential ecological risk. DP posed very high potential ecological risk. Integrated analysis reveals a distinctive ‘high-mercury anomaly at a distant location’ at the DP site: despite being situated farthest from the mercury mine, this region exhibits the most severe soil mercury concentrations and pollution levels. This anomalous spatial distribution is primarily driven by a compound pollution mechanism resulting from coordinated multi-mineral extraction activities. In this process, mercury, as an associated element during the development of sand, iron, lead-zinc, and other mineral resources, is continuously released into the environment through tailings leaching and particulate dispersion, ultimately leading to its significant accumulation in soils.

### 3.2 Bacterial diversity in Hg-contaminated soils

**Figure 3:**
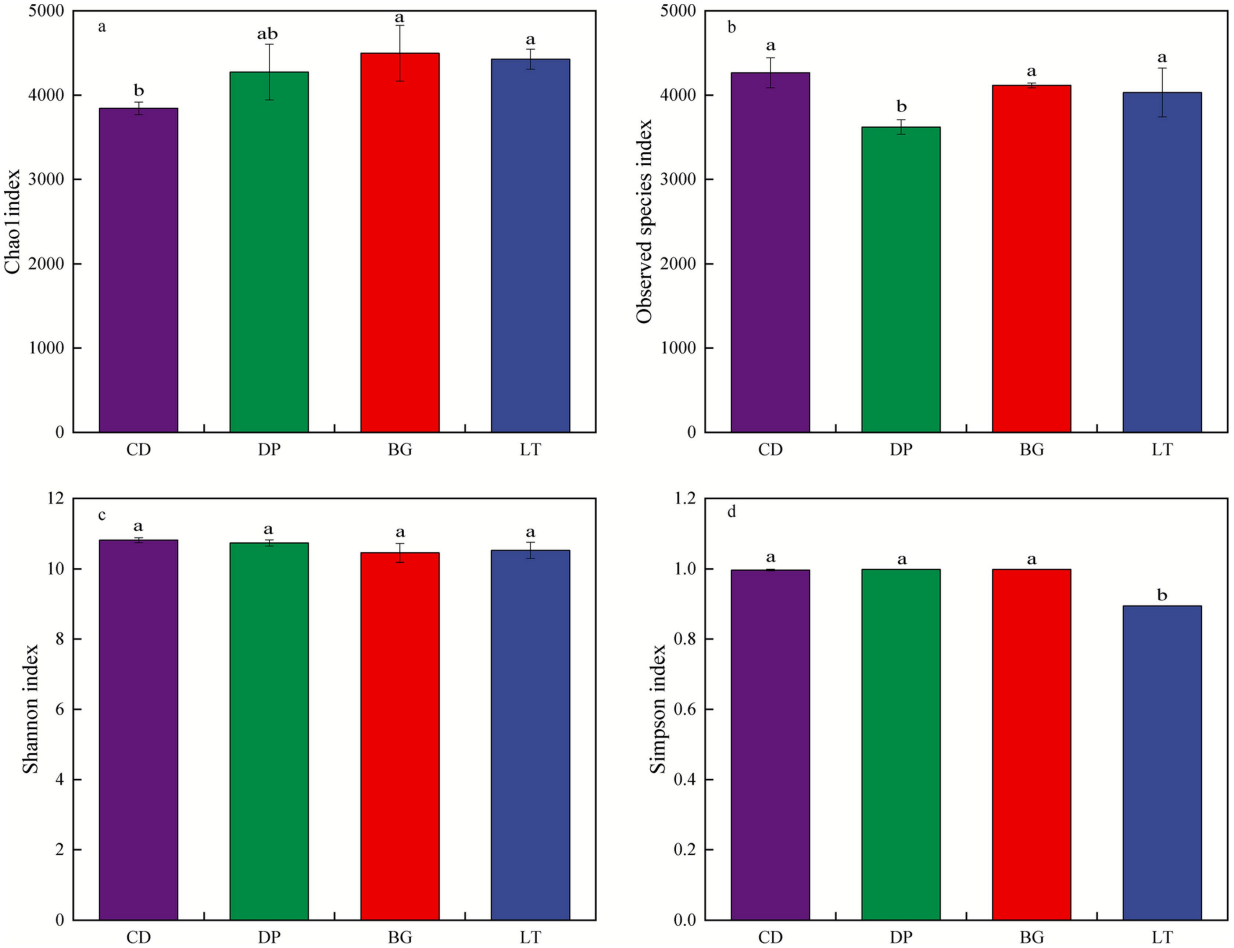
Alpha diversity indices of the soil bacterial community. **(a)** Chao1 index, **(b)** Observed species index, **(c)** Shannon index, **(d)** Simpson index.

Figure 3 shows the alpha diversity of the soil bacterial community. The Good’s coverage of each sample was greater than 99%, indicating that the probability of gene sequences being detected in the soil samples was high and that the sampling was essentially reasonable and could truly and effectively reflect the bacterial community of the soil samples. The Chao1 index was significantly greater in BG than in CD but was not significantly different from those in DP and LT (*p* < 0.05). The Observed species index was significantly greater in CD than in DP but was not significantly different from those in BG and LT (*p* < 0.05). The Simpson index was significantly lower in LT than in CD, DP and BG (*p* < 0.05). The differences in the Shannon index among the four regions were not significant (*p* < 0.05).

### 3.3 Analysis of bacterial community composition

A total of 19 phyla, 83 classes, 330 orders, 714 families, and 2,657 genera of bacteria were detected in the collected soil samples. On the basis of the annotation results, the results were statistically analyzed for the taxonomic units of the selected phyla and the lowest taxonomic unit (genus) at which most sequences could be annotated.

**Figure 4:**
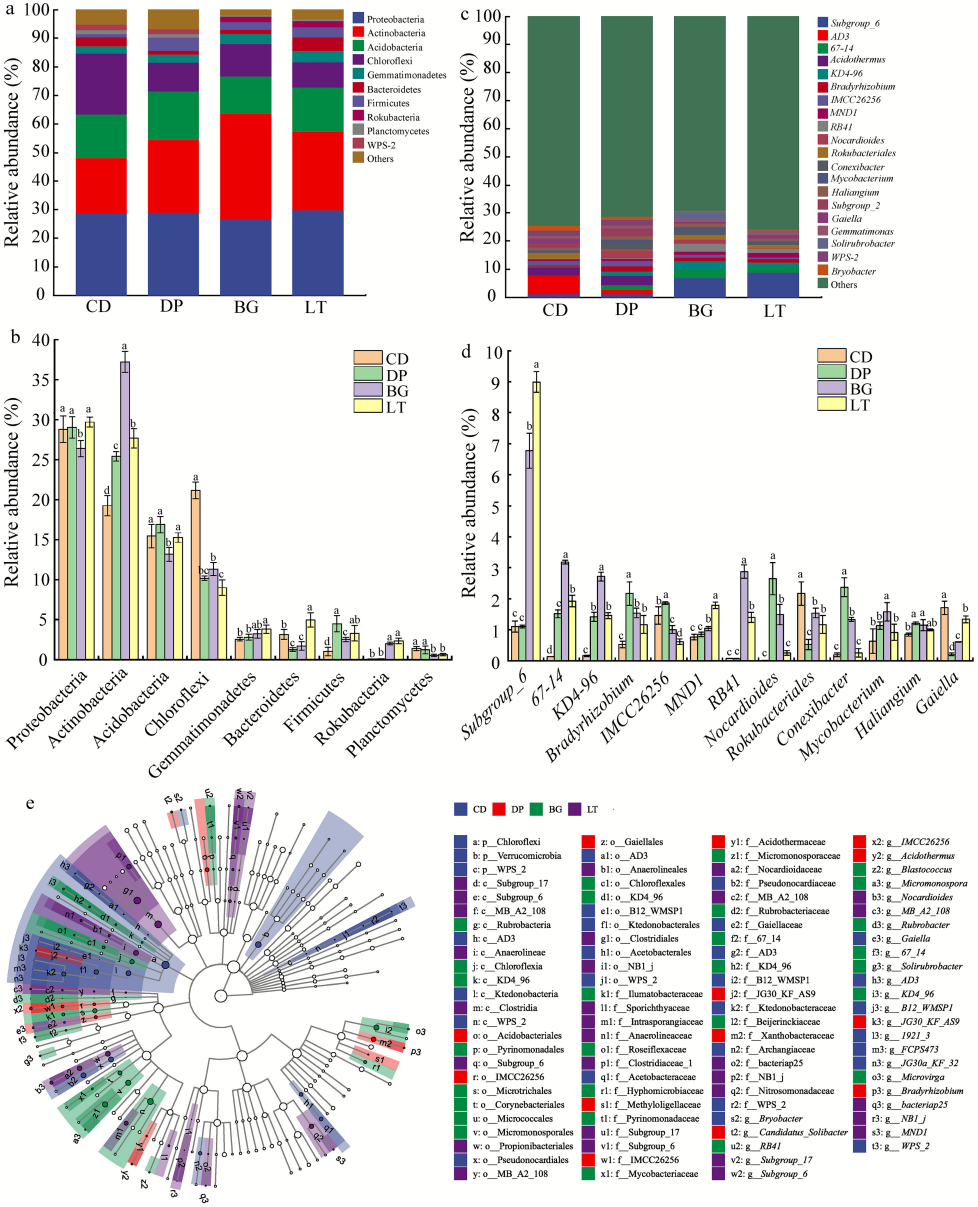
Soil bacterial community structure at the phylum level **(a,b)** and at the genus level **(c,d)** in different study areas. **(e)** LEfSe analysis of the soil bacterial communities. P: phylum; c: class; o: order; f: family; g: genus.

At the phylum level, the dominant bacterial flora were Proteobacteria, Actinobacteria, Acidobacteria, Chloroflexi, Gemmatimonadetes, Bacteroidetes, Firmicutes, Rokubacteria, and Planctomycetes (average relative abundance ≥ 1%) (Figure 4a). There was variability in the relative abundance of dominant soil bacterial phyla among the different regions (Figure 4b). Among them, the abundance of Chloroflexi was significantly greater in CD than in DP, BG and LT (*p* < 0.05); the abundance of Planctomycetes in CD was significantly greater than those in BG and LT (*p* < 0.05). The abundance of Acidobacteria was significantly greater in DP than in BG (*p* < 0.05); the abundance of Firmicutes was significantly greater in DP than in CD and BG (*p* < 0.05). The abundance of Actinobacteria was significantly greater in BG than in CD, DP and LT (*p* < 0.05). The abundance of Proteobacteria in LT was significantly greater than that in BG (*p* < 0.05). The abundances of Gemmatimonadetes and Rokubacteria in LT were significantly greater than those in BG and DP; the abundance of Bacteroidetes was significantly greater in LT than that in CD, DP and BG (*p* < 0.05).

At the genus level, the dominant bacterial flora were *Subgroup_6*, *67–14*, *KD4-96*, *Bradyrhizobium*, *IMCC26256*, *MND1*, *RB41*, *Nocardioides*, *Rokubacteriales*, *Conexibacter*, *Mycobacterium*, *Haliangium*, and *Gaiella* (average relative abundance ≥1%) (Figure 4c). There was variability in the relative abundance of dominant soil bacterial flora among the different regions (Figure 4d). Among them, the abundances of *Bradyrhizobium*, *IMCC26256*, *Nocardioides*, and *Conexibacter* were significantly greater in DP than in CD, BG and LT (*p* < 0.05); the abundance of *Haliangium* in DP was significantly greater than that in CD (*p* < 0.05). The abundances of *67–14*, *KD4-96*, *RB41*, and *Mycobacterium* were significantly greater in BG than in CD, DP and LT (*p* < 0.05). The abundances of *Subgroup_6* and *MND1* were significantly greater in LT than in CD, DP and BG (*p* < 0.05). The abundances of *Rokubacteriales* and *Gaiella* were significantly greater in CD than in DP, BG and LT (*p* < 0.05).

Because some key genera might be missed when dominant genera are screened at a threshold of average relative abundance ≥ 1%, LEfSe analysis was used to further screen significant differential species (i.e., key genera) of soil bacteria in the different study areas, and the results are shown in Figure 4e. A total of 98 differential bacterial species were identified by LEfSe analysis, 28, 13, 28, and 29 of which were identified in CD, DP, BG, and LT, respectively. At the genus level, excluding unidentified species, the differential species in CD soil mainly included *Bryobacter* and *Gaiella*. The differential species in DP soil mainly included *Candidatus_Solibacter*, *Acidothermus*, and *Bradyrhizobium*. The differential species in BG soil mainly included *Blastococcus*, *Micromonospora*, *Rubrobacter*, *Solirubrobacter*, and *Microvirga*. The differential species in LT soil was *Nocardioides*. These findings indicate that there are some significant differences in the species of soil bacteria in the different study areas.

### 3.4 Analysis of bacterial symbiotic networks

**Figure 5:**
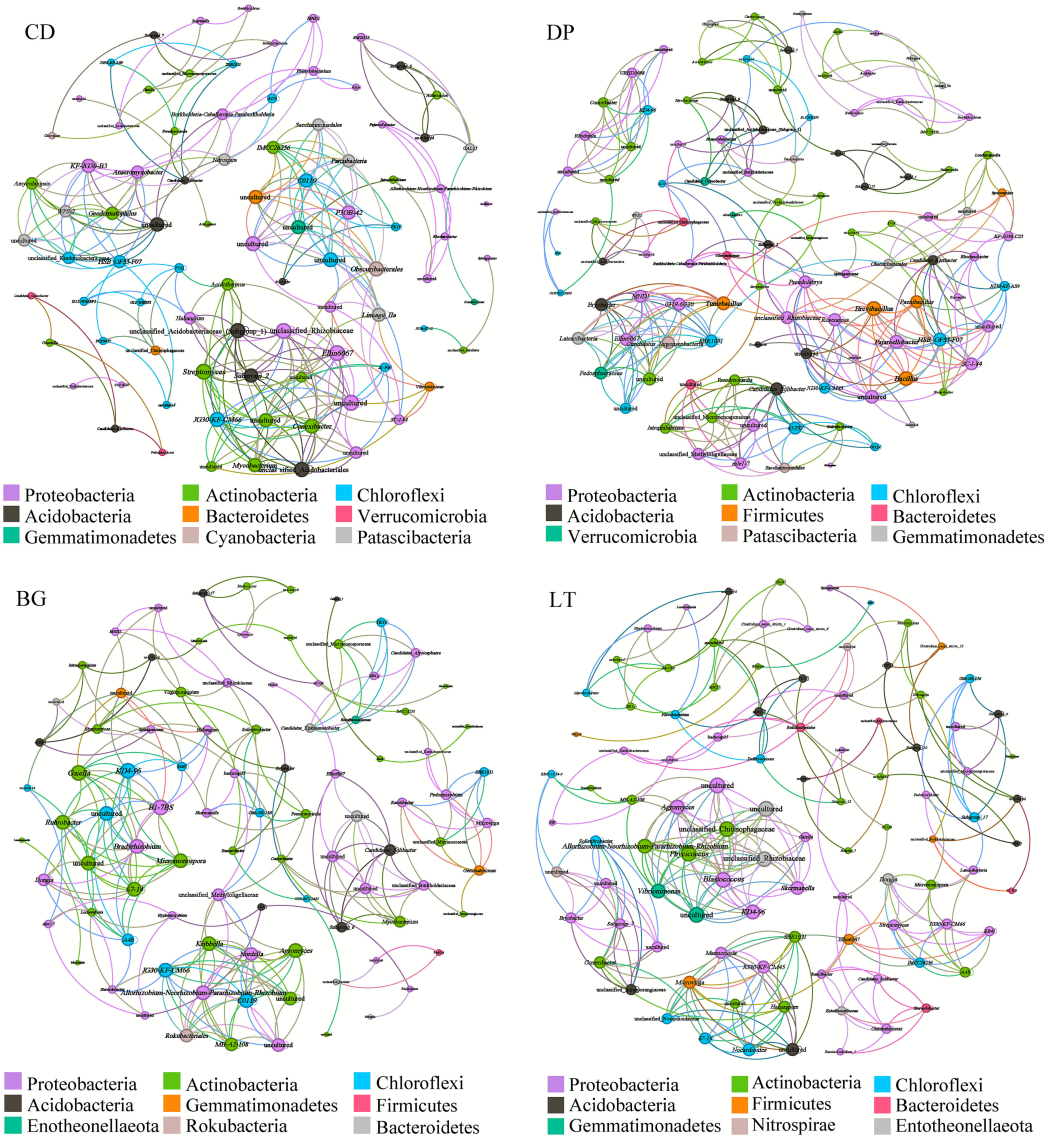
Co-occurrence network of soil bacterial communities.

Figure 5 shows that the dominant phyla involved in constructing the bacterial symbiotic network varied with study area. In the CK soil, Proteobacteria, Actinobacteria and Acidobacteria were the key taxa involved in the construction of the symbiotic network. In the BG, LT and DP soils, Proteobacteria, Actinobacteria, and Chloroflexi dominated the symbiotic network. This result indicates that the degree of involvement of bacterial communities in building symbiotic networks varied across the study area. Table 2 shows that the average degree, average clustering coefficient, modularity index, total nodes, and total edges in the DP soil microbial co-occurrence network had the highest values among the different regions, but the network diameter, graph density, and average path length were the lowest, indicating that compared with those in CD, BG and LT, DP soil bacteria community had a more complex network structure. In addition, the positive edges of the LT co-occurrence network had the highest value, but the negative edges had the lowest value, indicating that the network stability of the LT soil bacterial community was high. Table 2 illustrates that the interactions between soil microorganisms in different regions were all positively correlated, i.e., the interactions were mainly synergistic and cooperative, with weak competitive relationships, and the proportion of synergistic and cooperative microbial relationships was the highest in LT soils, which further indicated that the LT soil microbial network was most closely linked.

**Table 2:** Major topological properties of the soil fungal communities in the study areas.

Network properties	CD	DP	BG	LT
Average degree	7.82	7.873	7.225	7.58
Network diameter	19	15	20	16
Graph density	0.089	0.072	0.078	0.077
Average clustering coefficient	0.826	0.829	0.814	0.827
Average path length	6.179	4.919	5.716	5.306
Modularity index	0.755	0.830	0.812	0.821
Total nodes	89	110	94	100
Total edges	348	433	341	379
Positive edges/%	62.64	65.36	64.81	68.87
Negative edges/%	37.36	34.64	35.19	31.13

### 3.5 Bacterial metabolic functions in Hg-contaminated soils

**Figure 6:**
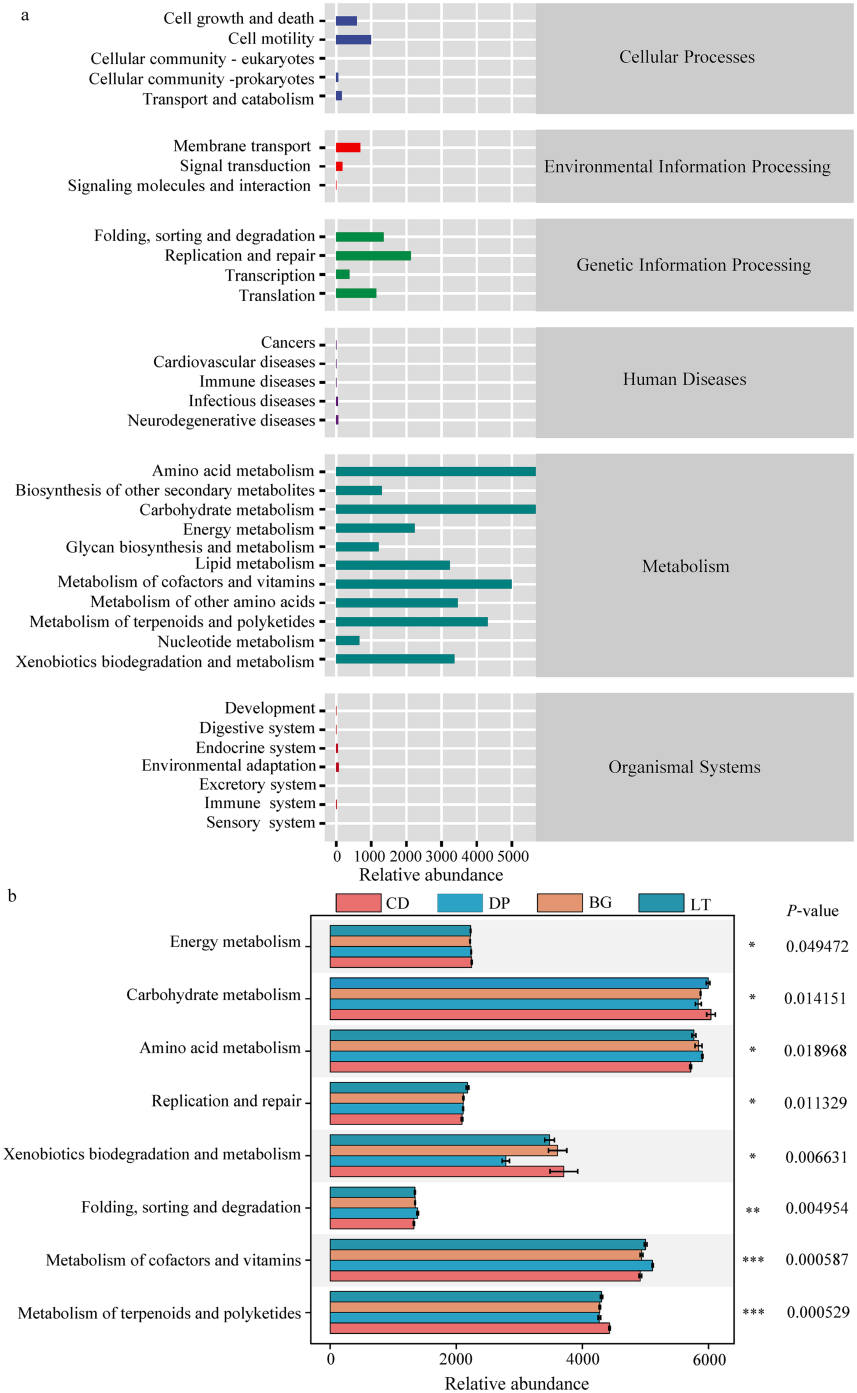
**(a)** KEGG functional pathway prediction and classification of all identified functional genes. **(b)** STAMP analysis of the abundance of functional genes at KEGG level 2. * Indicates a significant difference at the 0.05 level, ** indicates a significant difference at the 0.01 level, and *** indicates a significant difference at the 0.001 level.

The predicted functions of soil bacteria in each region were classified and analyzed with PICRUST2 software on the basis of the level 1 relative abundance and level 2 relative abundance via the KEGG database (Figure 6a). At level 1, a total of 6 biometabolic pathways were identified: (a) cellular processes, (b) environmental information processing, (c) genetic information processing, (d) human diseases, (e) metabolism, and (f) organismal systems. Among them, metabolism had the highest relative abundance, accounting for 82.20%. At level 2, a total of 35 biometabolic pathways were identified from the four study areas, and the top 10 functions in terms of relative abundance included 8 classes of metabolism and 2 classes of genetic information processing. These pathways included (a) carbohydrate metabolism, (b) amino acid metabolism, (c) metabolism of other amino acids, (d) metabolism of terpenoids and polyketides, (e) xenobiotic biodegradation and metabolism, (f) energy metabolism, (g) replication and repair, (h) folding, sorting and degradation, (i) lipid metabolism, and (j) metabolism of cofactors and vitamins. STAMP analysis (Figure 6b) revealed that ‘folding, sorting and degradation,’ ‘replication and repair,’ and ‘metabolism of cofactors and vitamins’ were significantly greater in LT than in CD, DP and BG (*p* < 0.05). Amino acid metabolism was significantly greater in BG than in DP and LT (*p* < 0.05). Carbohydrate metabolism was significantly greater in CD than in DP and LT (*p* < 0.05); energy metabolism was significantly greater in CD than in BG (*p* < 0.05); metabolism of terpenoids and polyketides was significantly greater in CD than in DP, BG and LT (*p* < 0.05); and xenobiotic biodegradation and metabolism were significantly greater in CD than in LT (*p* < 0.05). ‘Lipid metabolism’ and ‘metabolism of other amino acids’ were not significantly different among the four study areas (*p* < 0.05).

### 3.6 Soil factors affecting the functioning of soil bacterial communities

**Figure 7:**
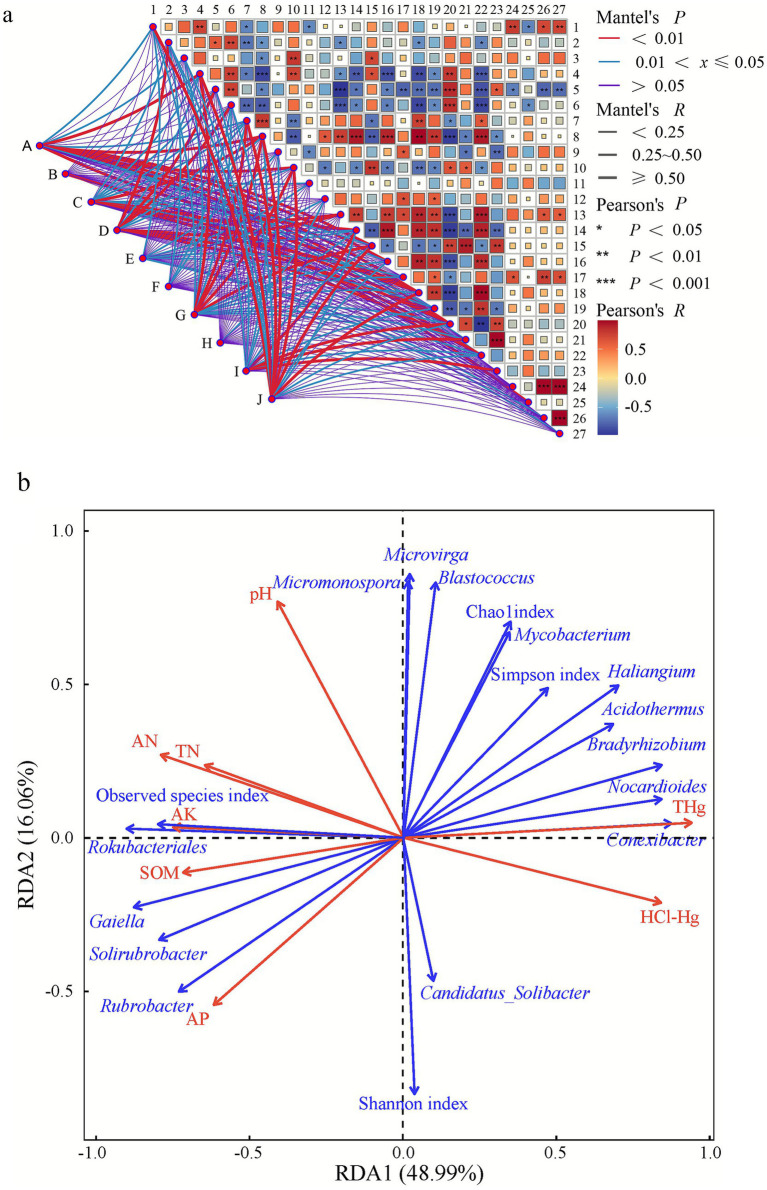
KEGG correlation analysis **(a)** and RDA **(b)** of the relationships between soil bacterial community function and soil properties. (A) Folding, sorting, and degradation; (B) Replication and repair; (C) Amino acid metabolism; (D) Carbohydrate metabolism; (E) Energy metabolism; (F) Lipid metabolism; (G) Metabolism of cofactors and vitamins; (H) Metabolism of other amino acids; (I) Metabolism of terpenoids and polyketides; (J) Xenobiotics biodegradation and metabolism. 1, pH value; 2, SOM; 3, TN; 4, AN; 5, AP; 6, AK; 7, HCl-Hg; 8, THg; 9, Chao1 index; 10, Observed species index; 11, Shannon index; 12, Simpson index; 13, *Acidothermus*; 14, *Bradyrhizobium*; 15, *Rokubacteriales*; 16, *Nocardioides*; 17, *Mycobacterium*; 18, *Conexibacter*; 19, *Haliangium*; 20, *Gaiella*; 21, *Solirubrobacter*; 22, *Bryobacter*; 23, *Rubrobacter*; 24, *Micromonospora*; 25, *Candidatus_Solibacter*; 26, *Blastococcus*; 27, *Microvirga.*

Figure 7a depicts the correlation of soil bacterial community functions with physicochemical factors and key bacterial genera. ‘Folding, sorting and degradation’ and ‘xenobiotic biodegradation and metabolism’ were significantly correlated with pH, TN, AN, HCl-Hg, THg, Observed species index, *Bradyrhizobium*, *Rokubacteriales*, *Nocardioides*, *Conexibacter*, *Gaiella*, and *Bryobacter*. ‘Folding, sorting and degradation’ was significantly correlated with *Acidothermus* and *Haliangium*.’ Xenobiotic biodegradation and metabolism’ was significantly correlated with AK. ‘Replication and repair’ was significantly correlated with *Candidatus_Solibacter*. ‘Amino acid metabolism’ and ‘metabolism of cofactors and vitamins’ were significantly correlated with AN, AK, HCl-Hg, THg, Observed species index, *Bradyrhizobium*, *Rokubacteriales*, *Nocardioides*, *Conexibacter*, *Gaiella*, and *Bryobacter*. ‘Amino acid metabolism’ was significantly correlated with SOM, *Acidothermus*, and *Haliangium*. ‘Metabolism of cofactors and vitamins’ was significantly correlated with pH, TN, and *Solirubrobacter*. ‘Carbohydrate metabolism’ was significantly correlated with SOM, AP, AK, HCl-Hg, THg, *Acidothermus*, *Bradyrhizobium*, *Conexibacter*, *Gaiella*, and *Bryobacter*. ‘Energy metabolism’ was significantly correlated with pH, the Chao1 index, the Shannon index, Mycobacterium, and *Haliangium*. ‘Lipid metabolism’ was significantly correlated with SOM and HCl-Hg. ‘Metabolism of other amino acids’ was significantly correlated with the Simpson index. ‘Metabolism of terpenoids and polyketides’ was significantly correlated with the Simpson index, SOM, AP, the Chao1 index, *Acidothermus*, *Bradyrhizobium*, *Rokubacteriales*, *Haliangium*, *Gaiella*, *Solirubrobacter*, *Bryobacter*, and *Rubrobacter*.

RDA was used to further analyse the relationships between soil bacterial community functions and soil physicochemical factors and key bacterial genera, and the results are shown in Figure 7b and Supplementary Table S1. The first two axes explained 65.05% of the variation in soil bacterial community functions, and the first and second axes explained 48.99 and 16.06%, respectively. Among them, soil physicochemical properties pH, THg and the bacterial community factor *Gaiella* were the major factors affecting soil bacterial community functions, explaining 72.83% (*p* = 0.001), 84.64% (*p* = 0.001), and 81.97% (*p* = 0.001) of the variation, respectively. In contrast, conventional nutrient factors (SOM, AP, AN, AK) show significantly lower explanatory power (ranging from 50.72 to 66.99%), though they maintain important modulating roles. These findings indicate that in mercury-contaminated ecosystems, the synergistic effects of heavy metal stress, soil pH, and specific mercury-tolerant microbial taxa are the dominant forces shaping microbial functional patterns, while conventional nutrient factors primarily play auxiliary regulatory roles.

## 4 Discussion

### 4.1 Bacterial diversity in Hg-contaminated soils

Soil microbial diversity is an important bioindicator of ecosystem health, exhibiting complex responses to heavy metal contamination that depend on both contaminant concentration and edaphic factors (Bhaduri et al., 2022). Our investigation revealed concentration-dependent phenomenon in mercury-contaminated soils: while severe Hg contamination consistently reduces bacterial diversity (Frossard et al., 2017), moderate contamination elicits a more nuanced response - increasing species diversity while decreasing species richness (Hyun et al., 2021). This apparent paradox likely reflects ecological restructuring, with Hg stress simultaneously eliminating sensitive taxa while promoting niche differentiation among tolerant species (Markowicz et al., 2016). Critically, these metal effects are modulated by soil biogeochemistry (Sazykin et al., 2023). We identified nitrogen availability (TN/AN content) as a key positive regulator of bacterial richness, which is consistent with the findings of Naylor et al. (2022), reflecting the dual role of nitrogen as a structural component of microbial cells and a metabolic driver of proliferation (Soumare et al., 2023). Conversely, the significant negative correlation between Shannon diversity and soil pH (Figure 6) highlights the role of pH as an environmental filter, a finding that is consistent with that of Yang et al. (2019) but contrasts with that of Cho et al. (2016). This pH-dependent response, particularly in our acidic CD/DP sites, demonstrates how suboptimal pH conditions can disrupt membrane integrity and protein stability, ultimately limiting microbial diversity (Zhou et al., 2024). Taken together, these findings highlight the multifaceted interplay between heavy metal toxicity and soil physicochemical properties in shaping microbial community dynamics.

### 4.2 Characterization of bacterial community structure in Hg-contaminated soils

Soil microbial communities serve as sensitive bioindicators of ecosystem health, with distinct phylum-level distributions reflecting environmental gradients across study sites (Naseem et al., 2024). Du et al. (2025) identified Proteobacteria, Acidobacteria, and Bacteroidetes as the dominant phyla in agricultural soils near the Wanshan mercury mining area, whereas Liu et al. (2014) reported predominant microbial compositions of Proteobacteria, Chloroflexi, Actinobacteria, and Acidobacteria in comparably affected areas. In this study, LEfSe analysis of agricultural systems located farther from the mining area identified Acidobacteria, Proteobacteria, and Actinobacteria as the key phyla. Despite partial overlap with previous findings, the distinct compositional patterns observed highlight the selective effects of spatial heterogeneity in soil physicochemical properties on microbial community assembly. Further integration with soil environmental factors revealed clear niche partitioning among these key taxa. Acidobacteria thrived in acidic DP soils (pH-driven selection of acidophilic taxa), Proteobacteria dominated nutrient-rich LT environments, and Actinobacteria flourished in the BG’s neutral-alkaline (pH 7.5 ~ 8.0) high-nutrient conditions. These patterns align with known ecological strategies, while Acidobacteria characterize oligotrophic systems, both Proteobacteria and Actinobacteria represent eutrophic taxa that drive critical ecosystem processes including nutrient cycling and organic matter decomposition (Mosley et al., 2022; Xie et al., 2024). Our findings provide microbial ecological evidence that the interaction between soil pH and nutrient availability serves as a key driver shaping the spatial patterning of microbial communities under heavy metal stress.

At the genus level, our analysis identified 12 key bacterial genera exhibiting distinct adaptation strategies to Hg contamination (Figure 4), which can be categorized into three functional groups based on their response patterns: resistant (*Rokubacteriales*, *Gaiella*, *Solirubrobacter*), tolerant (*Conexibacter*, *Bryobacter*, *Acidothermus*, *Bradyrhizobium*, *Nocardioides*, *Haliangium*), and neutral (*Micromonospora*, *Blastococcus*, *Microvirga*). The resistant group presented significant negative correlations with HCl-Hg or THg, suggesting active avoidance mechanisms, whereas the tolerant genera presented positive correlations, indicating metabolic adaptation to the presence of Hg. Notably, these metal-adapted communities maintain critical ecosystem functions despite contamination stress. *Bradyrhizobium*, known for its nitrogen-fixing ability and ability to promote plant growth even under arsenic stress (Pan et al., 2023; Salmi and Boulila, 2021), coexists with beneficial *Nocardioides* and *Rubrobacter*, which enhances crop disease resistance (Meena et al., 2020; Mousa et al., 2024) while demonstrating resistance to arsenic and antimony (Qiao et al., 2024). The community also includes specialized degraders such as the cellulolytic *Acidothermus* (Su et al., 2024) and the multifunctional *Solirubrobacter* and *Gaiella*, which participate in nutrient cycling while serving as potential PAH contamination indicators (Jara-Servin et al., 2024; Zhang et al., 2023). Of particular interest is the co-occurrence of beneficial taxa with metal-resistant pathogens such as *Mycobacterium* (Thomas et al., 2020), illustrating the complex reorganization of microbial interactions under chronic Hg exposure. The prevalence of multiple metal-adapted genera, including *Rokubacteriales, Bryobacter*, *Candidatus_Solibacter*, and *Halingium* in heavy metal-contaminated (Cu, Pb, Zn, Cr and As) soils (Belykh et al., 2024; Zhou et al., 2024) and Hg-Cu-Cd-Cr-resistant *Conexibacter* (Naseem et al., 2024) demonstrates the microbiome’s remarkable adaptive capacity. Network analysis revealed a shift from competitive to cooperative interactions in the restructured community (Figure 5 and Table 2), with synergistic relationships predominating among the soil bacterial communities. This reorganization, characterized by a decrease in sensitive species and enrichment of metabolically versatile, metal-tolerant taxa, highlights the microbial community’s resilience in maintaining ecosystem functioning under long-term heavy metal contamination conditions. The distinct metal resistance profiles of these genera, ranging from *Rokubacteriales’* Hg-specific resistance to *Conexibacter’s* broad metal tolerance, provide valuable insights for developing microbial indicators of soil pollution and informing strategies for the bioremediation of heavy metal-contaminated environments.

### 4.3 Influence of soil characteristics on soil bacterial community functions

As a critical determinant of soil health and ecosystem functioning, microbial functional diversity is highly sensitive to heavy metal contamination, serving as both a bioindicator of environmental stress and a mediator of biogeochemical processes (Nikitin et al., 2022). The complex interplay between heavy metals and the soil microbiota manifests through multiple pathways: direct toxicity to microbial cells, alteration of soil physicochemical properties, and subsequent restructuring of community composition and metabolic potential (Agarwal et al., 2024). Our findings align with those of Hoffland et al. (2020) and Nannipieri et al. (2017), confirming that spatial heterogeneity in fundamental soil parameters—particularly organic matter content, pH, and nutrient availability—sculpts microbial community structure and function through the formation of distinct ecological niches. Building on this foundation, our study systematically reveals functional metabolic resilience in soil microorganisms inhabiting areas distant from mining operations: despite mercury stress, carbohydrate metabolism and amino acid metabolism pathways maintained high activity, collectively accounting for 26.43% of the total predicted functions. This finding not only extends Bérard et al.'s (2014) insights on cadmium-induced metabolic restructuring to mercury-contaminated systems, but also demonstrates the capacity of microbial communities to sustain ecosystem stability by preserving core metabolic functions. RDA revealed three pivotal factors governing bacterial community function in Hg-contaminated soils: soil pH (a master regulator of microbial ecology), THg, and *Gaiella* (Figure 6b). These factors operate through interconnected mechanisms, with pH influencing metal bioavailability and nutrient solubility while modulating enzymatic activity (Naz et al., 2022). THg emerged as the dominant explanatory variable (84.64% contribution), exerting selective pressure through both direct toxicity and indirect community restructuring. Notably, certain bacterial taxa have evolved sophisticated resistance strategies, such as *Gaiella*’s production of metal-chelating extracellular polysaccharides (Salmi and Boulila, 2021) and *Streptococcus*’s pH-mediated metal precipitation (Zhang et al., 2025), highlighting the evolutionary arms race between microbes and metal stressors.

At the molecular level, heavy metals impose selective pressures that reshape microbial genomes, favor genes involved in metal resistance (e.g., genes encoding metallothioneins, metallotransporters and permeases), while disrupting metabolic gene networks (Nnaji et al., 2024). This genomic restructuring manifests distinct functional signatures, as evidenced by the positive correlations between specific taxa and metabolic pathways: *Bradyrhizobium* and *Rokubacteriales*, with xenobiotic degradation; *Haliangium*, with energy metabolism; and *Gaiella*, with cofactor/vitamin metabolism. The environmental filters of pH and Hg concentration create complex selection gradients, promoting metal-tolerant genera (*Micromonospora*, *Blastococcus*) while suppressing sensitive taxa (*Candidatus_Solibacter*, *Nocardioides*). Crucially, our analysis revealed how metal-induced community restructuring cascades through ecological networks, with cooperative symbioses (e.g., *Rokubacteriales*-*Rubrobacte*r) and competitive exclusions (e.g., *Bradyrhizobium*-*Gaiella*) collectively shaping metabolic potential. These interspecies interactions, coupled with the impact of Hg on nutrient cycling through the modulation of SOM, AN, and AK, create feedback loops that further constrain microbial functional diversity. The resulting metabolic trade-offs between survival strategies and biogeochemical functions underscore the precarious balance of microbial communities under metal stress, positioning functional diversity as both a sentinel of soil health and a predictor of ecosystem resilience in contaminated environments. These insights have significant implications for the development of targeted bioremediation strategies that address both metal toxicity and functional restoration in soil ecosystems.

## 5 Conclusion

This comprehensive study investigates mercury-contaminated agricultural soils across a distance gradient from China’s Tongren mining region, revealing a complex interplay between heavy metal pollution and soil microbial ecosystems. Our findings demonstrate that the agricultural soils in the study area have some degree of Hg contamination. Hg contamination was “light” to “moderate,” with potential ecological risks ranging from “moderate” to “high”-creating significant ecological and food safety risks. Microbial community analysis revealed distinct restructuring under Hg stress, with Acidobacteria, Proteobacteria and Actinobacteria emerging as the dominant phyla, while metal resistance genera, including *Rokubacteriales*, *Gaiella*, and *Solirubrobacter*, exhibited adaptive survival strategies. Functional profiling indicates that microbial communities maintain critical metabolic processes, particularly carbohydrate metabolism and amino acid metabolism pathways, despite contamination, with these two pathways collectively accounting for 26.43% of all predicted functions. Crucially, we identified the soil pH, total Hg content and specific bacterial taxa (notably *Gaiella*) as the primary drivers of microbial community function, independently explaining 72.83, 84.64, and 81.97% of the variance, respectively.

This investigation provides new scientific perspectives by redirecting research focus from mining-proximal zones to more distant agricultural soils. Our findings reveal that microbial communities in these remote areas maintain remarkable functional resilience through preserved core metabolic pathways and predominance of specific metal-tolerant taxa. The observed adaptive patterns suggest potential distinctions from microbial response mechanisms documented in heavily contaminated core mining areas, thereby offering novel insights into microbial ecological evolution across pollution gradients. These discoveries significantly advance our understanding of microbial adaptation mechanisms in contaminated soils and establish a scientific foundation for developing precision bioremediation approaches that simultaneously address metal detoxification and restoration of ecological functions across extensive agricultural systems.

## Data Availability

The raw data supporting the conclusions of this article will be made available by the authors, without undue reservation.

## Footnotes

1
http://www.Arb-silva.De
