# Supplementary material for: Mercury contamination alters soil microbial communities and functional traits in farmland soils of a mining region, south-western China
Source: Front Microbiol. 2025 Dec 18;16:1721310. doi: 10.3389/fmicb.2025.1721310 (PMC12756583; doi:10.3389/fmicb.2025.1721310)
Supplement: Supplementary file 1 [file Data_Sheet_1.docx]

**Table S1** RDA of the relationships between soil bacterial community function and soil properties

|  | Factors | Explained variance | *P* | Order |
| --- | --- | --- | --- | --- |
| Physicochemical factors | pH | 72.83 | 0.001 | 9 |
|  | SOM | 50.72 | 0.042 | 23 |
|  | TN | 45.54 | 0.077 | 25 |
|  | AN | 66.99 | 0.010 | 11 |
|  | AP | 65.13 | 0.008 | 13 |
|  | AK | 54.20 | 0.025 | 21 |
| Hg contents | HCl-Hg | 71.69 | 0.002 | 10 |
|  | THg | 84.64 | 0.001 | 1 |
| Microbial diversity indices and key bacterial genera | Chao1index | 59.12 | 0.017 | 17 |
|  | Observed species index | 61.64 | 0.017 | 14 |
|  | Shannon index | 66.98 | 0.009 | 12 |
|  | Simpson index | 46.24 | 0.050 | 24 |
|  | *Acidothermus* | 60.74 | 0.016 | 16 |
|  | *Bradyrhizobium* | 76.83 | 0.003 | 6 |
|  | *Rokubacteriales* | 81.23 | 0.001 | 3 |
|  | *Nocardioides* | 52.37 | 0.043 | 22 |
|  | *Mycobacterium* | 56.99 | 0.019 | 20 |
|  | *Conexibacter* | 76.88 | 0.001 | 5 |
|  | *Haliangium* | 74.07 | 0.001 | 8 |
|  | *Gaiella* | 81.97 | 0.001 | 2 |
|  | *Solirubrobacter* | 74.11 | 0.005 | 7 |
|  | *Rubrobacter* | 78.42 | 0.006 | 4 |
|  | *Micromonospora* | 57.48 | 0.023 | 19 |
|  | *Candidatus_Solibacter* | 18.20 | 0.416 | 26 |
|  | *Blastococcus* | 57.94 | 0.025 | 18 |
|  | *Microvirga* | 60.90 | 0.018 | 15 |
